# Survival after wedge resection versus lobectomy for stage IA second primary NSCLC with previous lung cancer-directed surgery

**DOI:** 10.3389/fonc.2022.890033

**Published:** 2022-08-10

**Authors:** Congkuan Song, Zilong Lu, Donghang Li, Shize Pan, Ning Li, Qing Geng

**Affiliations:** Department of Thoracic Surgery, Renmin Hospital of Wuhan University, Wuhan, China

**Keywords:** second primary lung cancer, second primary NSCLC, lobectomy, wedge resection, SEER

## Abstract

**Background:**

The surgical procedure for early-stage second primary non-small cell lung cancer (SP-NSCLC) remains controversial, especially for patients with previous lung cancer-directed surgery. This study aims to compare the survival after wedge resection and lobectomy for these patients.

**Methods:**

Stage IA SP-NSCLC patients with clear clinical information were searched from the Surveillance, Epidemiology, and End Results (SEER) database. The Cox proportional hazard model, the competing risk model, and the Kaplan–Meier survival curve were used to describe the survival difference between wedge resection and lobectomy. A 1:1 propensity score matching (PSM) method was also performed to reduce the potential impact of confounding factors between the two groups.

**Results:**

Of the 320 eligible stage IA SP-NSCLC patients included in this study, 238 (74.4%) patients underwent wedge resection and 82 (25.6%) patients received lobectomy. The 5-year overall survival (OS) was 61.3% with wedge resection and was 66.1% with lobectomy. Both before and after PSM, wedge resection showed similar OS and lung cancer-specific mortality as lobectomy in the entire cohort. Additionally, in all subgroup analyses, wedge resection demonstrated equivalent survival to lobectomy. However, in the female, sublobectomy for the first primary lung cancer, and interval ≤ 24 months subgroups, wedge resection displayed a higher lung cancer-specific mortality than lobectomy (fine-gray test, all *p* < 0.05).

**Conclusion:**

Overall, wedge resection is comparable to lobectomy in OS for stage IA SP-NSCLC patients with previous lung cancer-directed surgery. Therefore, we believe that wedge resection may be sufficient for these patients, although, in some cases, wedge resection has a higher lung cancer-specific mortality rate than lobectomy.

## Introduction

With the advancement in early detection technology of lung cancer and the close postoperative follow-up of lung cancer patients, the detection rate of second primary lung cancer (SPLC) has been growing. The efficacy and safety of surgical treatment for second primary non-small cell lung cancer (SP-NSCLC) patients have also been demonstrated in several studies (1–7). For the patients with resectable early-stage NSCLC, lobectomy remains the accepted standard surgical procedure (8). However, with the continuous improvement of medical technology and treatment concept, the treatment of lung cancer has become individualized and standardized. Two “maxima”, namely, maximum removal of tumor and maximum preservation of normal lung tissue, have become the development direction of lung tumor surgery. For lung cancer patients with previous lung cancer-directed surgery, in addition to having less lung tissue, they would also have a higher risk of developing another new primary lung cancer than the general population (9). Clinically, it may be difficult to accurately identify the second tumor as primary, recurrent, or metastatic. Since 1975, when Martini and Melamed came up with the diagnostic criteria (10) for multiple primary lung cancer (MPLC), there have been more and more reports on MPLC. In the SEER database, there is also a dataset on multiple primary events. The relevant information can be available to us from this website (https://seer.cancer.gov/tools/mphrules/). As with the Martini–Melamed criteria (10), SEER also considers tumor histology, location, and time since initial diagnosis to determine multiple primary events. However, there are still no clear treatment guidelines and plans for MPLC. For patients with SPLC with previous lung cancer-directed surgery, especially for patients who are elderly, have severe cardiopulmonary diseases, or are unwilling to undergo surgery, the limited residual lung tissue makes them more cautious when they face the surgical removal of the second tumor lesion. Radiotherapy, especially stereotactic radiotherapy, may be a good option for such patients (11). Compared with surgical resection, stereotactic radiotherapy has the advantages of non-invasive treatment, the immediate recovery of activity after treatment, and the treatment of multiple lesions simultaneously. However, everything has two sides. Radiotherapy-related toxicities, such as bronchial stenosis, necrosis, and esophageal ulcers, have increased the concern (12). Surgery, to our knowledge, is still currently the only treatment offering potential cure and long-term survival in patients with SPLC. Despite significant breakthroughs in the diagnosis and treatment of lung cancer, it has not been determined whether lobectomy has a better survival advantage than wedge resection for patients with early-stage SP-NSCLC, especially those with a history of radical surgery for the first primary lung cancer (FPLC). A recent SEER-based study (13) has reported that SPLC demonstrated similar survival with lobectomy and wedge resection. However, the study did not delve into the differences in survival between the two procedures in various specific circumstances. Faced with the complexity of patients’ clinical situation, further stratified analysis is still necessary. Thus, combining with a variety of methods, this study compared the survival after lobectomy and wedge resection using overall survival (OS) and lung cancer-specific mortality as outcomes.

## Materials and methods

### Data source and patient selection

**Figure 1:**
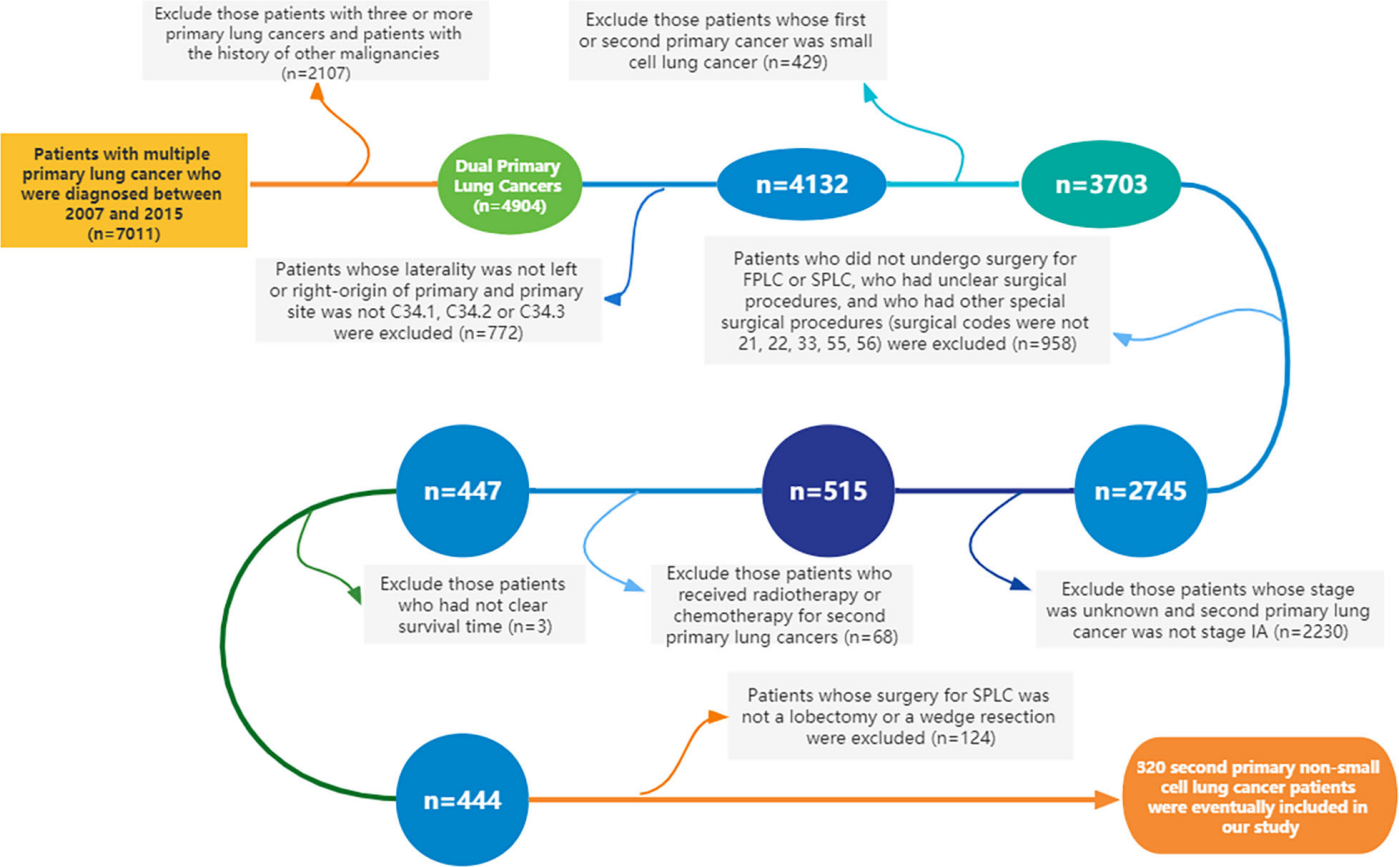
Flowchart of the inclusion and exclusion criteria for the cases in this study (FPLC, first primary lung cancer; SPLC, second primary lung cancer).

Using SEER*Stat 8.3.5 software (http://seer.cancer.gov/seerstat/), we extracted data from the SEER database, which was open to the public for research purposes. A total of 320 patients with dual primary non-small cell lung cancers were extracted from the SEER database. Patients meeting the following criteria were included in this study: ① Year of diagnosis was between 2007 and 2015. ② Site and morphology. Site recoded ICD-O-3/WHO 2008: Lung and Bronchus. ③ Events (1 of 124 selected for display): lung and bronchus. Furthermore, cases with three or more primary lung cancers and whose first or second primary cancer was small cell lung cancer (SCLC) were excluded from this study. Cases without clear status, survival time, and American Joint Committee on Cancer (AJCC) stage were also removed. The specific inclusion and exclusion criteria, as well as the case screening process of this study, are detailed in **Figure 1**. The 8th edition of the TNM staging system was applied in the present study. The collected variables included age at diagnosis, sex, “race record”, “primary site-labeled”, laterality, “ICD-O-3 Hist/behav, malignant”, tumor size, “months since index” (the time interval between two primary tumors), AJCC Stage, “COD to site recode”, “Rx Sumn-Surg Prim Site (1998+)”, “radiation record”, and “chemotherapy record”.

### Description of surgery types and histological types

In this study, sublobectomy includes wedge resection (SEER surgery codes: 21) and segmental resection (SEER surgery codes: 22). Lobectomy means lobectomy with mediastinal lymph node dissection (SEER surgery codes: 33) and pneumonectomy contains complete pneumonectomy, sleeve pneumonectomy, standard pneumonectomy, total pneumonectomy, and resection of whole lung (SEER surgery codes: 55) and with mediastinal lymph node dissection (radical pneumonectomy) (SEER surgery codes: 56). Other surgery codes were excluded in our study. The detailed SEER surgery codes can be viewed from the website (https://seer.cancer.gov/manuals/2018/AppendixC/Surgery_Codes_Lung_2018.pdf#search=surgery%20lung).

In addition, by the following International Classification of Diseases for Oncology histology codes, we categorized histology into three groups: ① adenocarcinoma (8140, 82508255, 8260, 8310, 8323, 8480–8481, 8550, and 8574); ② squamous cell carcinoma (8070–8074 and 8083); ③ other NSCLCs (8012–8013, 8020, 8022, 8033, 8046, 8230, and 8246). The present study did not include the histological type of SCLC (8041 and 8045), either FPLC or SPLC.

### Statistical analysis

In this study, categorical variables were represented by number (percentage), and continuous variables were expressed as mean (standard deviation, SD). The Chi-square test or Fisher’s exact test was used to compare differences in baseline characteristics, as appropriate. The Cox proportional hazard model, the competitive risk model, and the Kaplan–Meier survival curve were used to describe the survival difference between wedge resection and lobectomy. Statistical comparisons between survival curves were performed with the log-rank test. To reduce the influence of confounding factors, we performed propensity score matching (PSM) for some important clinical factors including age at the SPLC, sex, race, histology of SPLC, tumor distribution of two lesions, grade of SPLC, interval between two lesions, surgery types of FPLC, AJCC stage of FPLC, tumor size of SPLC, radiation for FPLC, and chemotherapy for FPLC. PSM was performed using the “nearest” method with a caliper of 0.05 to reduce the potential impact of confounding factors between the two groups. The “MatchIt” package in R was adopted to calculate propensity scores. In addition, subgroup analysis was also performed to better characterize the prognostic differences between the two operations (wedge resection and lobectomy). All analyses were performed in version 3.6.0 of R software. A *p*-value of less than 0.05 was accepted for statistical significance.

## Results

### Patient characteristics

Following the detailed screening procedures in **Figure 1**, 320 patients with stage IA SP-NSCLC diagnosed from 2007 to 2015 were eventually included in this study. Wedge resection (*n* = 238, 74.4%) was performed on significantly more patients than lobectomy (*n* = 82, 25.6%). The detailed clinicopathological features are shown in **Table 1**. Of the 320 patients included, 190 were female and 130 were male; 68.95% (131/190) of the female patients and 82.31% (107/130) of the male patients underwent wedge resection for the second NSCLC. The average age of patients with lobectomy and wedge resection was similar, 68.34 ± 8.48 and 68.44 ± 9.05 years, respectively. Adenocarcinoma was predominant in the lobectomy group and wedge resection group (72.0% and 72.7%, respectively). Wedge resection was performed in the overwhelming majority of patients (45/52) when the two lesions were located in the ipsilateral different lobes. There were 259 patients with two lesions in the bilateral, and only 27.03% (70/259) chose lobectomy for the second lesion. Additionally, it could also be observed that regardless of the surgical procedures (sublobectomy, lobectomy, or pneumonectomy) performed on the first lesion, wedge resection was performed on the second lesion significantly more than lobectomy. In the lobectomy group, patients with a tumor diameter of less than 10 mm were the least, accounting for 14.6%. In the wedge resection group, patients with a tumor diameter ranging from 20 to 30 mm were the least, accounting for 10.9%.

**Table 1:** Characteristics of patients in the wedge resection group and the lobectomy group before PSM.

Characteristics	Subgroups		Lobectomy, *n* (%)	Wedge resection, *n* (%)	*p*-value
**Total**		—	82 (100%)	238 (100%)	
**Age (years)**		Continuous (mean, SD)	68.34 (8.48)	68.44 (9.05)	0.930
**Sex**		Female Male	59 (72.0) 23 (28.0)	131 (55.5) 107 (45.0)	0.011
**Race**		White Black Others	72 (87.8) 6 (7.3) 4 (4.9)	201 (84.5) 20 (8.4) 17 (7.1)	0.724
**Histology of SPLC**		Adenocarcinoma Squamous CC Other NSCLCs	59 (72.0) 21 (25.6) 2 (2.4)	173 (72.7) 50 (21.0) 15 (6.3)	0.317
**Tumor distribution**		Same lobe Ipsilateral different lobes Bilateral	5 (6.1) 7 (8.5) 70 (85.4)	4 (1.7) 45 (18.9) 189 (79.4)	0.014
**Grade of SPLC**		I well II moderate III/IV poor/undifferentiated Unknown	25 (30.5) 28 (34.1) 23 (28.0) 6 (7.3)	55 (23.1) 109 (45.8) 53 (22.3) 21 (8.8)	0.232
**Interval (months)**		Continuous (mean, SD)	36.15 (21.96)	32.86 (21.38)	0.234
**Surgery types of FPLC**		Sublobectomy Lobectomy Pneumonectomy	13 (15.9) 69 (84.1) 0 (0.0)	35 (14.7) 200 (84.0) 3 (1.3)	0.581
**AJCC stage of FPLC**		Stage I Stage II Stage III Stage IV	68 (82.9) 6 (7.3) 7 (8.5) 1 (1.2)	173 (72.7) 23 (9.7) 33 (13.9) 9 (3.8)	0.274
**Tumor size of SPLC**		<10 mm ≥10 mm, <20 mm ≥20 mm, ≤30 mm	12 (14.6) 50 (61.0) 20 (24.4)	86 (36.1) 126 (52.9) 26 (10.9)	<0.001
**Radiation for FPLC**		Unknown/No	81 (98.8)	225 (94.5)	0.191
		Yes	1 (1.2)	13 (5.5)	
**Chemotherapy for FPLC**		Unknown/No	80 (97.6)	228 (95.8)	0.698
		Yes	2 (2.4)	10 (4.2)	

### Survival after lobectomy versus wedge resection before and after PSM

**Figure 2:**
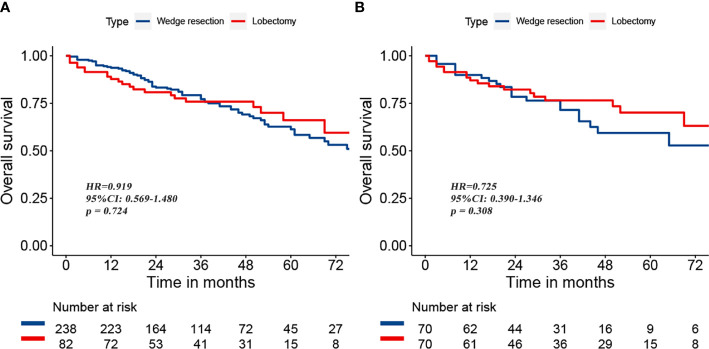
Overall survival after wedge resection versus lobectomy before **(A)** and after **(B)** PSM.

**Figure 3:**
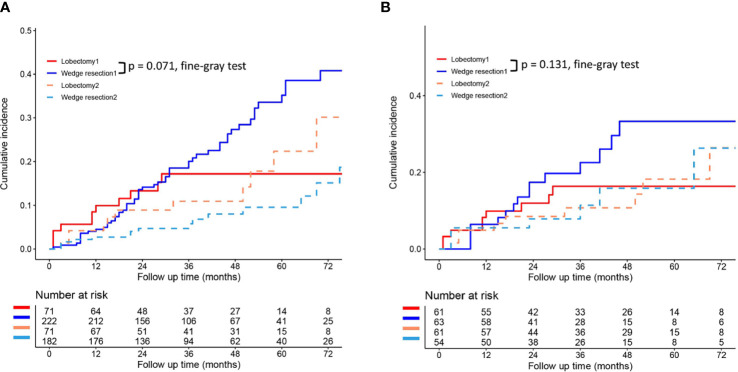
Lung cancer-specific mortality after wedge resection versus lobectomy before **(A)** and after **(B)** PSM. These figures show a comparison between the cumulative incidence of lung cancer death and that of other causes in the two surgical procedures (lobectomy and wedge resection). **Note:** “Lobectomy 1” refers to the cumulative incidence curve of such patients who underwent lobectomy and died of lung cancer. “Wedge resection 1” refers to the cumulative incidence curve of such patients who underwent wedge resection and died of lung cancer. “Lobectomy 2” refers to the cumulative incidence curve of such patients who underwent lobectomy and died from other causes. “Wedge resection 2” refers to the cumulative incidence curve of such patients who underwent wedge resection and died from other causes.

To reduce the potential impact of differences in clinical features between the lobectomy group and the wedge resection group on outcomes, we performed PSM. After 1:1 matching, the differences in clinical variables between the two groups were significantly reduced, as shown in **Table 2**. To further compare survival after lobectomy and wedge resection, OS and lung cancer-specific mortality were used as the main prognostic indicators. We found that OS after wedge resection and lobectomy were comparable both before (log-rank test, *p* = 0.724; **Figure 2A**) and after (log-rank test, *p* = 0.308; **Figure 2B**) PSM. In the entire cohort (*n* = 320), the 5-year OS was 61.3% with wedge resection and was 66.1% with lobectomy. The two were also comparable in lung cancer-specific mortality (fine-gray test, all *p* > 0.05), as shown in **Figure 3**.

**Table 2:** Characteristics of patients in the wedge resection group and the lobectomy group after PSM.

Characteristics	Subgroups		Lobectomy(No. , %)	Wedge resection(No. , %)	*p*-value
**Total**		—	70 (100%)	70 (100%)	
**Age (years)**		Continuous (mean, SD)	68.59 (8.18)	69.30 (9.01)	0.624
**Sex**		Female Male	49 (70.0) 21 (30.0)	52 (74.3) 18 (25.7)	0.706
**Race**		White Black Others	60 (85.7) 6 (8.6) 4 (5.7)	61 (87.1) 4 (5.7) 5 (7.1)	0.771
**Histology of SPLC**		Adenocarcinoma Squamous CC Other NSCLCs	50 (71.4) 18 (25.7) 2 (2.9)	52 (74.3) 16 (22.9) 2 (2.9)	0.925
**Tumor distribution**		Same lobe Ipsilateral different lobes Bilateral	1 (1.4) 6 (8.6) 63 (90.0)	4 (5.7) 7 (10.0) 59 (84.3)	0.366
**Grade of SPLC**		I well II moderate III/IV poor/undifferentiated Unknown	18 (25.7) 25 (35.7) 22 (31.4) 5 (7.1)	21 (30.0) 29 (41.4) 14 (20.0) 6 (8.6)	0.494
**Interval (months)**		Continuous (mean, SD)	35.10 (21.24)	36.67 (21.90)	0.667
**Surgery types of FPLC**		Sublobectomy Lobectomy Pneumonectomy	12 (15.7) 59 (84.3) 0 (0.0)	16 (22.9) 54 (77.1) 0 (0.0)	0.392
**AJCC stage of FPLC**		Stage I Stage II Stage III Stage IV	57 (81.4) 6 (8.6) 6 (8.6) 1 (1.4)	57 (81.4) 4 (5.7) 7 (10.0) 2 (2.9)	0.847
**Tumor size of SPLC**		<10 mm ≥10 mm, <20 mm ≥20 mm, ≤30 mm	10 (14.3) 45 (64.3) 15 (21.4)	11 (15.7) 45 (64.3) 14 (20.0)	0.960
**Radiation for FPLC **		Unknown/No	69 (98.6)	70 (100.0)	1.000
		Yes	1 (1.4)	0 (0.0)	
**Chemotherapy for FPLC**		Unknown/No	68 (97.1)	70 (100.0)	0.476
		Yes	2 (2.9)	0 (0.0)	

FPLC, first primary lung cancer; SPLC, second primary lung cancer; Squamous CC, Squamous cell cancer; NSCLC, non-small cell lung cancer.

### Survival comparison based on subgroup analysis

**Figure 4:**
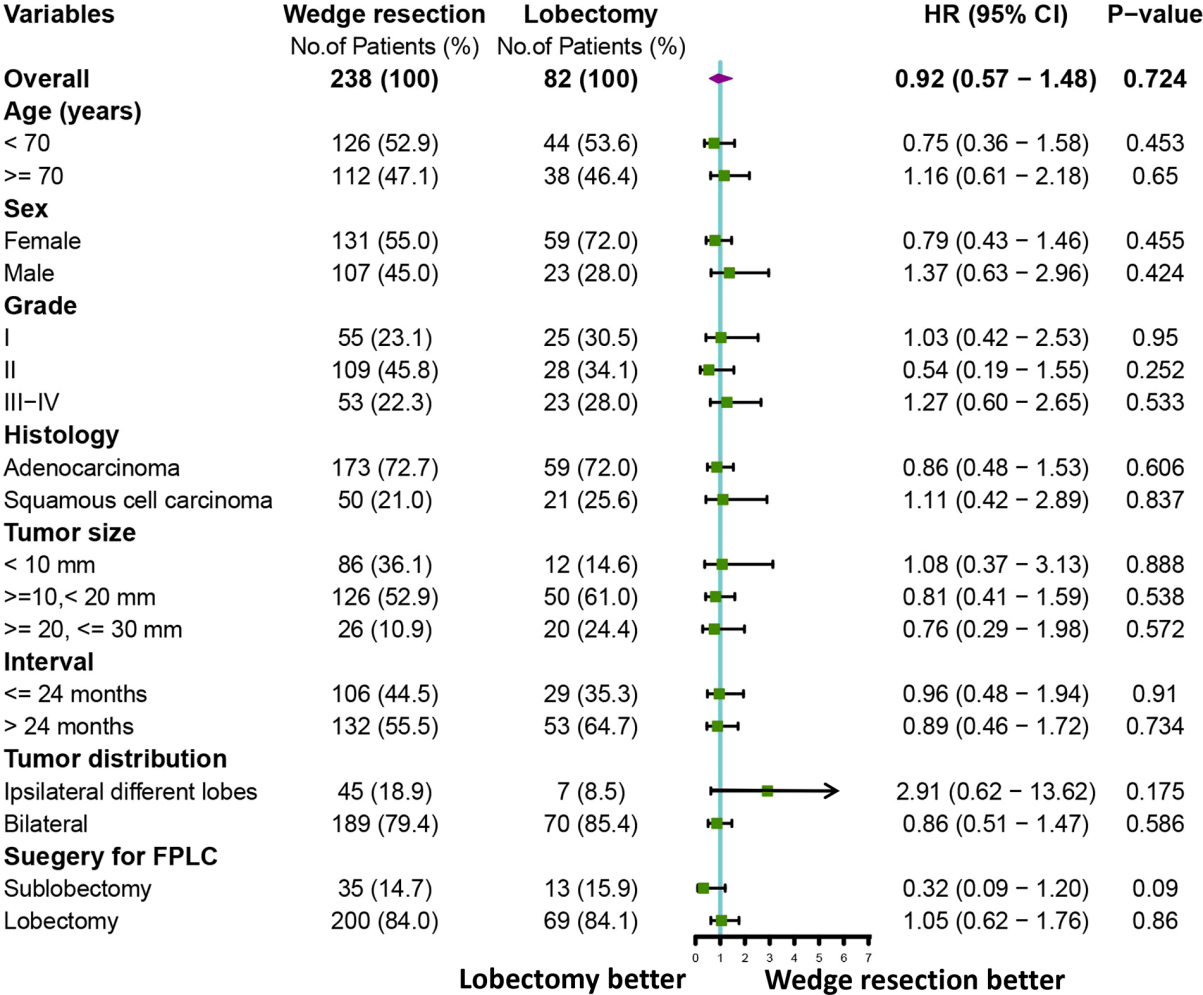
A forest plot showing the overall survival comparison between the two operations (lobectomy and wedge resection). Univariable Cox analysis and subgroup analysis were performed.

**Figure 5:**
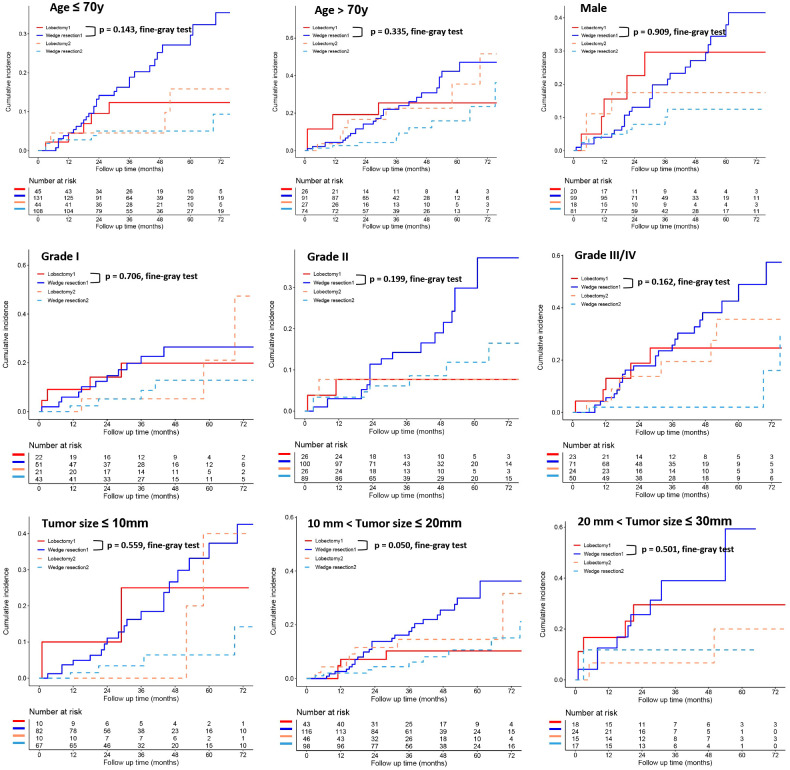
The differences in lung cancer-specific mortality between the two operations (lobectomy and wedge resection) based on subgroup analyses of factors with prognostic significance (such as age, sex, grade, and tumor size). These figures show a comparison between the cumulative incidence of lung cancer death and that of other causes in the two surgical procedures (lobectomy and wedge resection). **Note:** “Lobectomy 1” refers to the cumulative incidence curve of such patients who underwent lobectomy and died of lung cancer. “Wedge resection 1” refers to the cumulative incidence curve of such patients who underwent wedge resection and died of lung cancer. “Lobectomy 2” refers to the cumulative incidence curve of such patients who underwent lobectomy and died from other causes. “Wedge resection 2” refers to the cumulative incidence curve of such patients who underwent wedge resection and died from other causes.

**Figure 6:**
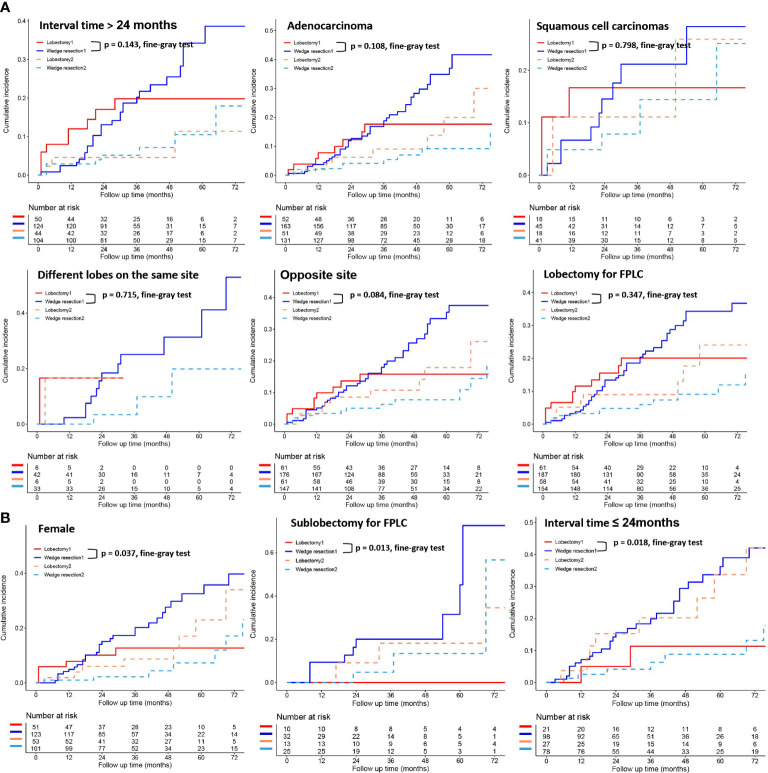
The differences in lung cancer-specific mortality between the two operations (lobectomy and wedge resection) based on subgroup analyses of factors with prognostic significance (such as interval time between the two primary lesions, histological types, location of the two primary lesions, the operation method for FPLC and sex). These figures show a comparison between the cumulative incidence of lung cancer death and that of other causes in the two surgical procedures (lobectomy and wedge resection). “Lobectomy 1” refers to the cumulative incidence curve of such patients who underwent lobectomy and died of lung cancer. “Wedge resection 1” refers to the cumulative incidence curve of such patients who underwent wedge resection and died of lung cancer. “Lobectomy 2” refers to the cumulative incidence curve of such patients who underwent lobectomy and died from other causes. “Wedge resection 2” refers to the cumulative incidence curve of such patients who underwent wedge resection and died from other causes.

To further clarify the survival after lobectomy and wedge resection in different clinical subgroups, we conducted subgroup analysis based on age, sex, histology, grade, tumor size, tumor distribution, time interval between two primary cancers, and type of first operation. We found that in all clinical subgroups, wedge resection demonstrated equivalent OS to lobectomy (all *p* > 0.05; **Figure 4**). Similarly, wedge resection and lobectomy had similar lung cancer-specific mortality in most clinical subgroups (**Figures 5**, **6A**). However, in the female, sublobectomy for FPLC, and interval ≤ 24 months subgroups, wedge resection had a higher lung cancer-specific mortality than lobectomy (fine-gray test, all *p* < 0.05; **Figure 6B**).

## Discussion

At present, there is no uniform standard for the surgical method of MPLC. Some scholars (14–16) have reported that lobectomy should be the first choice for the second primary tumor, followed by segment or wedge resection. However, sublobectomy, including segmentectomy and wedge resection, has also been studied as the main surgical method (3, 17). At the cost of damaging more pulmonary functional reserve compared with segmentectomy or wedge resection, lobectomy can remove more intrapulmonary lymph nodes and significantly reduce the recurrence rate of tumor (8), which can undoubtedly affect the postoperative quality of life of patients, especially those with a history of lung cancer-directed surgery. For stage IA SP-NSCLC patients with previous lung cancer-directed surgery, to date, it has not been fully understood whether lobectomy is more conducive to survival than wedge resection. Although several retrospective studies (18–20) and a recent SEER-based study (13) have found that lobectomy did not show a significant survival advantage over sublobectomy for SPLC patients, these studies still lacked an in-depth study on SPLC patients who have undergone radical surgery for primary lung cancer. To better solve this problem and provide references for further clinical research and practice, we carried out this study.

In the present study, wedge resection demonstrated equivalent OS to lobectomy, both before and after PSM. Similar results were observed in all clinical subgroups. Unlike the previous studies (4, 16, 17, 21), participants in this study were patients with stage IA SP-NSCLC with previous lung cancer-directed surgery. Moreover, the sublobectomy mentioned in their studies included not only wedge resection, but also other surgical methods such as segmental resection. To our knowledge, although wedge resection and segmentectomy were classified as sublobectomy, there were significant differences between them. Wedge resection was defined as non-anatomical resection of the lung parenchyma, and was not necessary to determine the histological structure of bronchus and pulmonary vessels, while segmentectomy was the anatomical resection of the lung parenchyma, and required the disconnection of segmental bronchus, segmental vessels, and segmental parenchyma. In view of the different anatomical pathways of wedge resection and segmentectomy, it was necessary to discuss the two surgical methods separately. Therefore, this study emphasized the difference in survival between wedge resection (rather than segmentectomy and/or wedge resection) and lobectomy. This study further confirmed that stage IA SP-NSCLC patients with previous lung cancer-directed surgery demonstrated similar OS with lobectomy and wedge resection.

In clinical practice, the relative location of two primary tumors had an extremely important influence on the final selection of surgical procedure. Generally speaking, when two tumor lesions were in the ipsilateral different lobes, lobectomy for the second lesion tended to lead to pneumonectomy, which was a risk factor affecting the prognosis of patients (4, 16, 20). This study found that when the two lesions were in the ipsilateral different lobes, wedge resection demonstrated equivalent OS to lobectomy. Thus, we believe that in this case, lobectomy for the second lesion may not result in a greater survival benefit than wedge resection. In agreement with our research, Ishigaki and his colleagues (4) suggested that if FPLC and SPLC were on the same side of the lung and FPLC received lobectomy, sublobectomy should be a priority for the SPLC. Additionally, surgical choice regarding the optimal extent of resection for a second primary tumor on the contralateral side is also controversial. Yang et al. (18) reported that a limited resection (sublobectomy) for the contralateral second tumor did not have a negative effect on OS in these patients with stage I bilateral MPLC. This was also confirmed by the findings of this study. Similarly, other previous studies (1, 16, 19, 20) did not demonstrate a significant difference in prognosis with respect to lobectomy versus sublobectomy for the second tumor. The choice of surgery for the second primary tumor is challenging for thoracic surgeons, especially for patients with a history of lobectomy or pneumonectomy. In addition to the maximum preservation of pulmonary functional reserve, maximum tumor resection is also an oncological principle to be followed. An adequate margin (>2 cm or the tumor diameter) is a prerequisite for sublobectomy. In a number of studies (19, 22), sublobectomy has been proved to have similar therapeutic effect to lobectomy for patients with early single primary lung cancer. Therefore, we believe that under strict patient screening criteria, sublobectomy including wedge resection is worthy of choice for thoracic surgeons.

Overall, this study provided evidence that wedge resection produced similar survival rate to lobectomy in stage IA SP-NSCLC patients with previous lung cancer-directed surgery, and wedge resection and lobectomy had similar lung cancer-specific mortality in most cases. A correct understanding of the difference in OS and lung cancer-specific mortality between the two surgical approaches might help clinicians make more reasonable choices.

This study still has the following limitations. First, much of the detailed information (such as imaging findings, pulmonary function index, and related basic diseases), which may be an important reference for clinicians to make treatment decisions, is not available in the SEER database. Second, the number of patients with stage IA SP-NSCLC included in this study was still relatively small, and the postoperative follow-up time was also short. Third, the nature of a retrospective study and the strict screening criteria in this study inevitably resulted in selection bias. Considering the deficiency of retrospective analysis, further prospective analysis is recommended.

In conclusion, wedge resection demonstrated equivalent survival to lobectomy in Stage IA second primary NSCLC patients with lung cancer-directed surgery. Considering the limitations of the present study, relevant prospective studies are still necessary.

## Data availability statement

The original contributions presented in the study are included in the article/supplementary material. Further inquiries can be directed to the corresponding author.

## Ethics statement

The SEER database is a public database with free access to data, so ethics committee approval is not required for this study. Written informed consent for participation was not required for this study in accordance with the national legislation and the institutional requirements.

## Author contributions

CS designed the study and reviewed relevant literature and drafted the manuscript. CS, ZL, and DL conducted all statistical analyses. All authors contributed to the article and approved the submitted version.

## Funding

This work was supported by grants from the National Natural Science Foundation of China (Nos. 81770095, 81700093, and 8210082163), the Fundamental Research Funds for the Central Universities (No. 2042021kf0081), and Science Fund for Creative Research Groups of the Natural Science Foundation of Hubei Province (No. 2020CFA027).

## Acknowledgments

This study used the Surveillance, Epidemiology, and End Results (SEER) database. We acknowledge the efforts of the National Cancer Institute in the creation of the database. We also thank Weidong Hu, Sheng Li, Zhiquan Wu, Donghu Yu, Zixin Guo, Qingwen Wang, Yujin Wang, and Jingyu Huang for our preliminary revision, review, and constructive comments. They have made great and selfless contributions to this paper.

## Conflict of interest

The authors declare that the research was conducted in the absence of any commercial or financial relationships that could be construed as a potential conflict of interest.

## Publisher’s note

All claims expressed in this article are solely those of the authors and do not necessarily represent those of their affiliated organizations, or those of the publisher, the editors and the reviewers. Any product that may be evaluated in this article, or claim that may be made by its manufacturer, is not guaranteed or endorsed by the publisher.

## Abbreviations

FPLC, first primary lung cancer; SPLC, second primary lung cancer; SP-NSCLC, second primary non-small cell lung cancer; MPLC, multiple primary lung cancer; OS, overall survival; SCLC, small cell lung cancer; AJCC, American Joint Committee on Cancer; ICD-O, International Classification of Diseases for Oncology; SEER, Surveillance, Epidemiology, and End Results.
